# A novel automated SARS-CoV-2 saliva PCR test protects a global asymptomatic workforce

**DOI:** 10.1038/s41598-021-92070-w

**Published:** 2021-06-16

**Authors:** Nikki Carter, Maryam Clausen, Rebecca A. Halpin, Colin Blackmore, Kang Cai, Oona Delpuech, Alexander Kohlmann, Otto Magnusson, Ruth March, Daniel O’Neill, Kasthuri Prakash, James Sherwood, Tabetha Sundin, Jason Swift, Azar Tarakameh, Marilou Wijdicks, Daniel Wise, Mark Fidock

**Affiliations:** 1 Biopharmaceutical Development, Biopharmaceuticals R&D, AstraZeneca, Gaithersburg, MD USA; 2 Discovery Sciences, Biopharmaceuticals R&D, AstraZeneca, Cambridge, UK; 3 Discovery Sciences, Biopharmaceuticals R&D, AstraZeneca, Gothenburg, Sweden; 4 Early Oncology, Oncology R&D, AstraZeneca, Cambridge, UK; 5 Early Oncology, Oncology R&D, AstraZeneca, Gaithersburg, MD USA; 6 Precision Medicine and Biosamples, Oncology R&D, AstraZeneca, Cambridge, UK; 7 Precision Medicine and Biosamples, Oncology R&D, AstraZeneca, Gaithersburg, MD USA; 8 Global Medical Affairs, Oncology Business Unit, AstraZeneca, Gaithersburg, MD USA; 9 R&D IT, AstraZeneca, Cambridge, UK

**Keywords:** Molecular medicine, Infection, Diagnosis

## Abstract

Regular PCR testing of nasopharyngeal swabs from symptomatic individuals for SARS-CoV-2 virus has become the established method by which health services are managing the COVID-19 pandemic. Businesses such as AstraZeneca have also prioritised voluntary asymptomatic testing to keep workplaces safe and maintain supply of essential medicines to patients. We describe the development of an internal automated SARS-CoV-2 testing programme including the transformative introduction of saliva as an alternative sample type.

## Introduction

**Figure 1:**
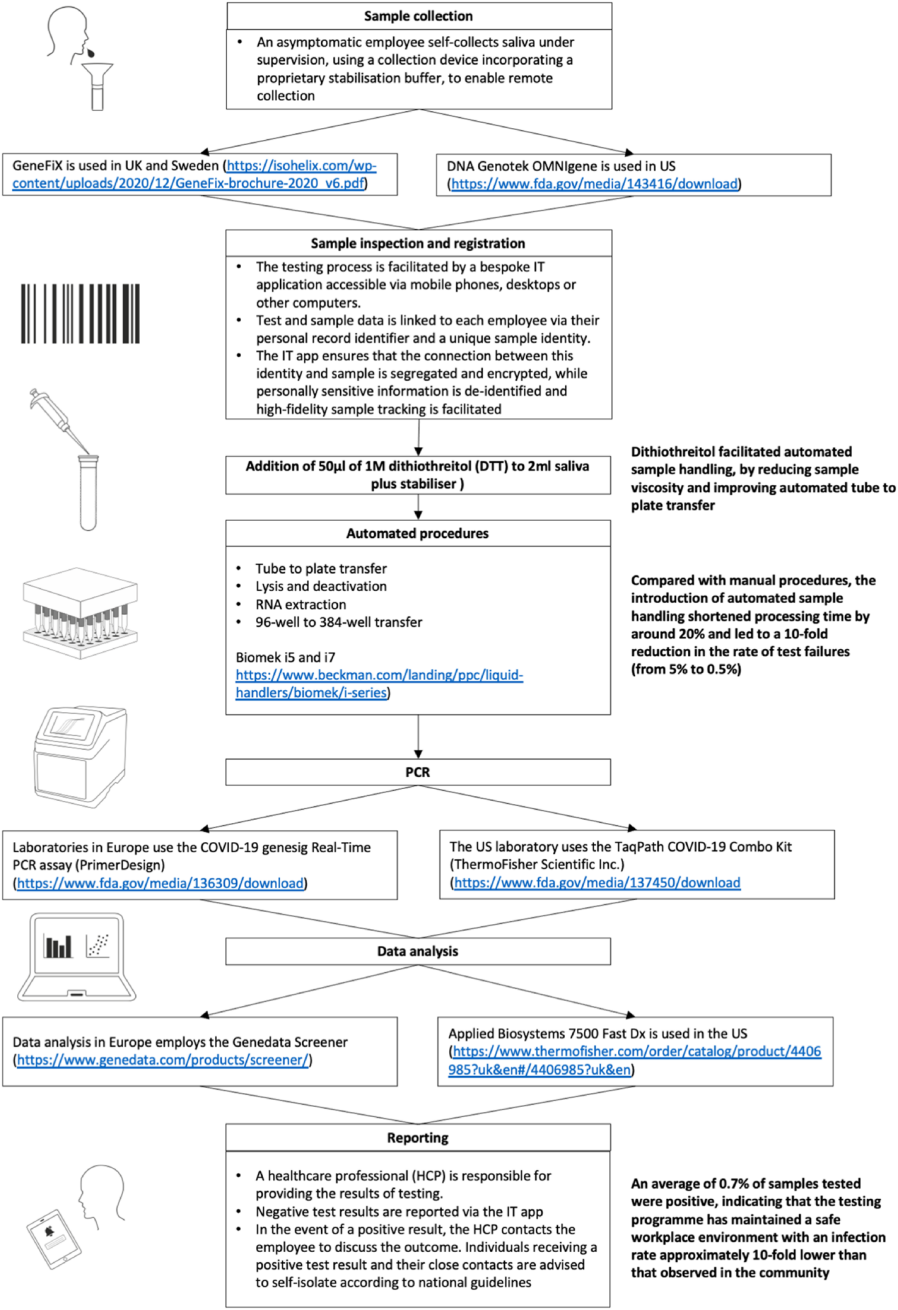
Overview of the automated workflow for SARS-CoV-2 testing of asymptomatic employees. Saliva samples are tested at one of three AstraZeneca laboratories: in Cambridge (UK), Gothenburg (Sweden) or Gaithersburg, MD (USA). The workflow was risk assessed and follows government guidance enabling the experimentation to be conducted at biosafety level 2 (BSL2), conforming to local laboratory standards including Good Clinical Practice (GCP) and Clinical Laboratory Improvement Amendments (CLIA) as appropriate.

Several organisations have responded to the challenge of keeping the workplace safe by implementing SARS-CoV-2 testing for asymptomatic individuals, either in their own laboratories or outsourced (https://www.gov.uk/government/publications/coronavirus-covid-19-testing-guidance-for-employers/annex-b-a-practical-guide-for-employers-who-want-to-offer-workplace-testing-for-asymptomatic-employees; https://blog.aboutamazon.co.uk/working-at-amazon/how-we-ramped-up-onsite-covid-19-testing-for-amazon-employees; https://www.bbc.co.uk/news/business-55370312). AstraZeneca has taken the additional step to increase adoption in a voluntary programme by operating automated SARS-CoV-2 testing, at scale and in an industrial context, using saliva as the sample type (Fig. 1).

**Figure 2:**
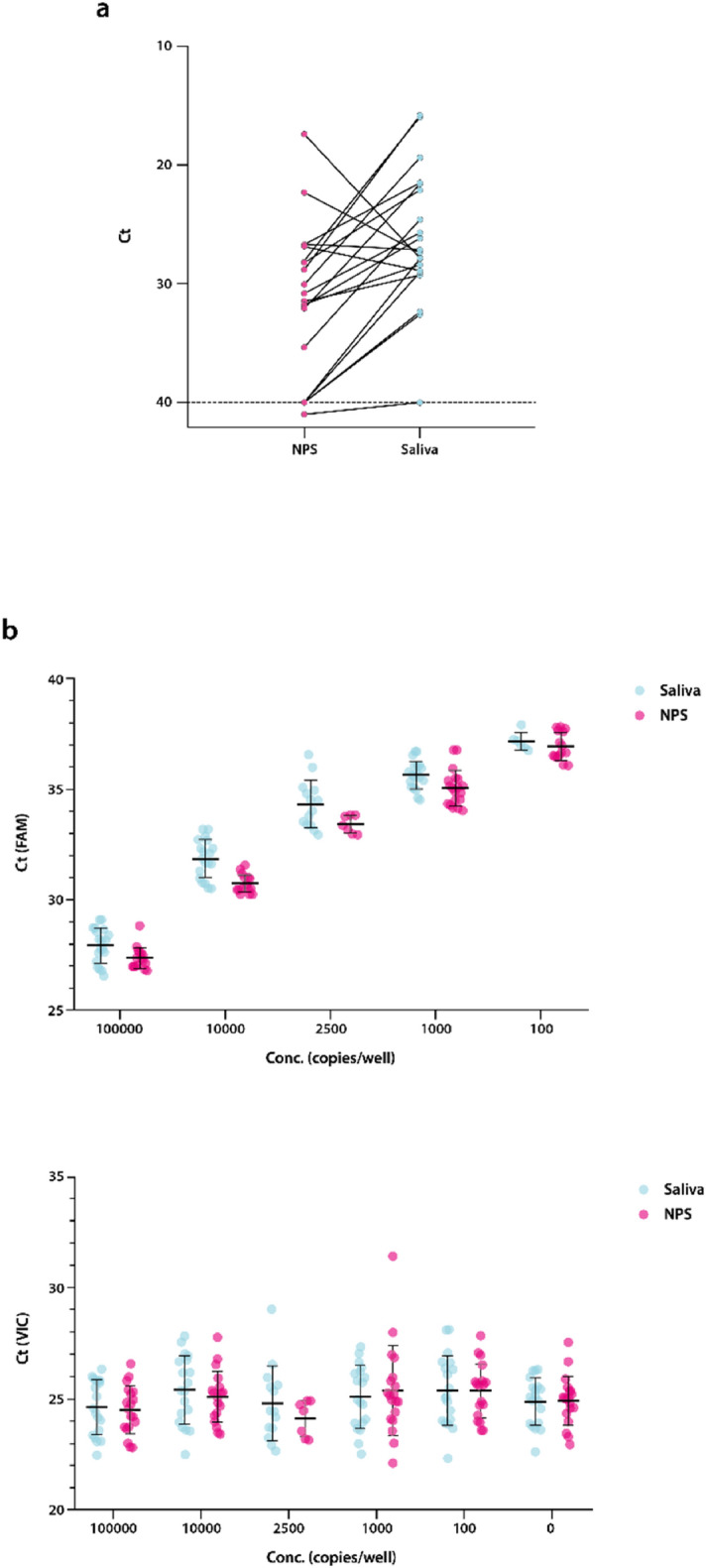
Clinical and analytical validation of the SARS-CoV-2 PCR tests enabling the transition from nasopharyngeal swabs (NPS) to saliva. (**a**) Analysis of 20 paired clinical saliva samples and NPS tested using the Taqpath RT-PCR COVID-19 combo kit, Thermo Fisher Scientific Inc. Cycle threshold (Ct) values for all samples/detectors were generated using the Design and Analysis Software version 2.4.3 (Thermo Fisher Scientific Inc), N gene data shown, with equivalent results seen using the genesig COVID-19 Real-Time PCR assay, PrimerDesign (PD) (data not shown). (**b**) Comparison of the analytical sensitivity of testing saliva samples and NPS, conducted on contrived samples using the PD assay. The sensitivity for detecting SARS-CoV-2 was similar for both sample types (FAM), and sample type had no effect on extraction or amplification (VIC).

In mid-March 2020, AstraZeneca set up an internal programme of voluntary SARS-CoV-2 testing for asymptomatic employees in the United Kingdom (UK), Sweden, and the United States of America (US) who could not work from home and were not able to obtain testing through their countries’ national efforts. Launched in 18 days, this programme was focused on workers responsible for maintaining the company’s supply of medicines, critical research and development laboratory staff and essential business employees. The initial launch of this effort used established nasopharyngeal swab (NPS) collection, supervised by a healthcare professional (HCP) (https://www.cdc.gov/coronavirus/2019-ncov/lab/guidelines-clinical-specimens.html). However, this proved to be uncomfortable and unpopular, leading to lower adoption than required. Following reports that saliva samples could be used in place of NPS for SARS-CoV-2 detection^1^ we evaluated and subsequently implemented saliva as our preferred sample type (Fig. 2a and b). By February 2021 approximately 70,000 SARS-CoV-2 PCR tests have been completed within our three internal global testing centres, of which 54,000 are based on saliva.

Following the introduction of saliva testing, adoption by employees increased approximately fourfold and over 90% of 1062 employees surveyed expressed a preference for the change^2^.

Asymptomatic employees follow a 5-step process for SARS-CoV-2 testing:

1. Employee requests a test via a bespoke IT application that includes the employee recording potential symptoms for COVID-19 and any other information required by local laws.
2. An HCP confirms that the individual is asymptomatic, has provided informed consent and orders the test.
3. Collection of the sample as advised by manufacturers’ and local health authorities’ instructions.
4. Analysis of sample for the presence of SARS-CoV-2 at one of three AstraZeneca laboratories: in Cambridge (UK), Gothenburg (Sweden) or Gaithersburg, MD (USA). The workflow was risk assessed prior to initiating employee testing and followed government guidance enabling the experimentation to be conducted at biosafety level 2 (BSL2), conforming to local laboratory standards including Good Clinical Practice (GCP) and Clinical Laboratory Improvement Amendments (CLIA) as appropriate. Positive results were reported at cycle threshold (Ct) values of ≤ 40 and following the manufacturer’s instructions.
5. Return of results by the HCP. Negative PCR results are reported in the IT app whereas the HCP contacts the employee to discuss the outcome of positive test results. The average turnaround time from sample receipt to result is < 24 h.

As expected, this testing cascade in asymptomatic individuals requires high analytical sensitivity. In our asymptomatic cohort, positive samples had a mean Ct value of 32 (Ct range of 40–15), where a Ct of 32 corresponds to a sample with a viral RNA load of approximately 1 × 10^5^ copies per mL, consistent with reported values in similar populations^3^. Interestingly, although higher Ct values are associated with asymptomatic samples, compared to samples from symptomatic cohorts, these differences are neither consistent nor statistically significant^4^. Recent publications have proposed that saliva collection reflects peak infection, predicts COVID-19 outcome and is associated with lower overall cost^5–7^ compared to nasopharyngeal collection.

This project was facilitated by a bespoke IT application accessible via mobile phones, desktops or other computers. Test and sample data is linked to each AstraZeneca employee via their personal record identifier and a unique sample identity. The IT app ensures that the connection between this identity and sample is segregated and encrypted, while personally sensitive information is de-identified and high-fidelity sample tracking is facilitated.

An average of 0.7% samples tested to date were positive in this voluntary internal programme, and individuals and their close contacts were advised to self-isolate according to national guidelines (https://www.ecdc.europa.eu/sites/default/files/documents/Guidance-for-discharge-and-ending-of-isolation-of-people-with-COVID-19.pdf; https://www.cdc.gov/coronavirus/2019-ncov/if-you-are-sick/quarantine.html). Overall, this programme has achieved its objective of maintaining a safe workplace environment by keeping the infection rate approximately tenfold lower than that observed in the respective communities.

## Methods

### Establishing asymptomatic testing

#### genesig COVID-19 Real-Time PCR (UK and Sweden)

AstraZeneca laboratories in Europe used the genesig COVID-19 Real-Time PCR assay (PrimerDesign) (PD) (https://www.fda.gov/media/136309/download), approved in Europe for use with NPS and saliva samples. This assay detects the ORF1ab gene and can detect 0.33 copies of whole viral genome RNA/µL, with a sensitivity of ≥ 95% and 100% specificity. The bioinformatic analysis of SARS-CoV-2 genomic epidemiology published on the GISAID EpiCoV database is reviewed weekly by PD to verify that the assay is effective for new variants.

The assay was verified for linearity, reproducibility, user, temporal and instrument variability at AstraZeneca laboratories. Assessments were performed using SARS-CoV-2 RNA contrived samples (Twist Synthetic SARS-CoV-2 RNA (Control 1 and Control 2), Twist Bioscience, https://www.twistbioscience.com/resources/product-sheet/twist-synthetic-sars-cov-2-rna-controls), demonstrating that the assay was performing within acceptable limits as laid out in the instructions for use (IFU).

Linearity was shown to be high, with 93% of 14 experiments meeting the slope acceptance criteria (1.0 ± 0.15) and 100% of 18 runs having an R^2^ ≥ 0.99 and meeting the acceptance criteria of ≥ 0.95. Limit of detection (LoD) was confirmed as 1 copy/µL in 98% (41/42) of replicates, which is suitable for viral detection in asymptomatic individuals.

Intra-inter assay precision experiments indicated that the assay detected the contrived samples consistently. When an average Ct from replicate plates (same RNA extraction) was used for classification, 100% detection was observed for dilutions from 20 to 200,000 copies of RNA per well across 4 days of testing.

#### TaqPath RT-PCR COVID-19 Combo Kit (Thermo Fisher Scientific Inc.) (USA)

In the AstraZeneca USA CLIA laboratory, the PD kit was not approved for use and available at the time of laboratory set up. Hence, assessments were performed using the TaqPath RT-PCR COVID-19 Combo Kit (ThermoFisher Scientific Inc.) (TF) (https://www.fda.gov/media/137450/download). The TF Kit has specific target sequences for 3 genes: ORF1ab, N Protein, S protein. According to the manufacturer, the LoD of the TF Kit is 10 copies of whole viral genome RNA, which will detect ≥ 95% positive samples.

Verification experiments used the same panel of Twist SARS-CoV-2 RNA contrived samples and successfully demonstrated assay linearity and dynamic range down to 10 copies per qRT-PCR reaction LoD. Reproducibility was demonstrated using spiked-in RNA level of 10 copies per reaction.

### Transition to saliva samples

#### Sample collection

Following a comparison of extraction and amplification efficiency for different saliva sample collection devices with appropriate regulatory approval, GeneFix (https://isohelix.com/wp-content/uploads/2020/12/GeneFix-brochure-2020_v6.pdf) was selected for use in Europe, and DNA Genotek OMNIgene (https://www.fda.gov/media/143416/download) in the US. These devices incorporate a funnel to facilitate saliva collection into a tube and contain a proprietary stabilisation buffer, enabling remote collection. Aligned to the instructions for use, employees were advised to be well hydrated and to fast for 30 min before sample collection^8^. Employees were supervised but self-collected their saliva samples^9^.

#### Sample handling

To simplify the protocol, the lysis buffer, proteinase K and the internal extraction control (IEC) could be premixed up to 2 h in advance of use and added to a saliva sample simultaneously without impairing stability or sensitivity of RNA extraction.

#### Clinical sensitivity

20 paired saliva samples and NPS specimens were obtained from patients determined to be positive for SARS-Cov-2 by a separate qRT-PCR assay (AllPlex 2019-nCOV, https://www.fda.gov/media/137178/download) performed no more than 3 days prior to collection. Analysis demonstrated that the saliva sample was positive in every matched pair in which the NPS was positive (Fig. 2a).

#### Analytical validity

The analytical validity of saliva testing was evaluated through a comparison with NPS testing for impact on extraction, amplification and sensitivity and SARS-CoV-2 detection using contrived samples (Twist Bioscience [UK and Sweden]; Qnostics SARS-CoV-2 Analytical Q Panel 01, Cat # SCV2AQP01-B [USA]).

The sensitivity for detecting SARS-CoV-2 was similar in contrived saliva and nasal samples for both assays used (Fig. 2b). In both cases assay failures were only seen at the lowest concentration and at comparable rates. Saliva did not impact extraction or amplification.

Interlaboratory assay concordance was monitored and maintained by the regular evaluation of the Qnostics SARS-CoV-2 Analytical Q Panel 01 [Cat # SCV2AQP01-B [USA]].

#### Optimisation of saliva testing

Saliva testing protocols were optimised as follows to ensure a low intrinsic test failure rate. This resulted in a failure rate of < 0.5% of samples per run.

(i) *Sample volume*
  Input volumes between 50 and 600 µL were evaluated, 600 µL limited RNA binding to the Beckman Coulter beads during extraction whereas 50 µL resulted in increased assay failures (https://www.beckman.com/search#q=C58529AA&t=coveo-tab-techdocs). The input volume for both PCR tests was standardised at 200 µL.
(ii) *Duration of reverse transcription*
  Extending the reverse transcription time to 30 minutes for saliva samples (N=8) reduced assay failure and improved sensitivity (Ct values), although the effect size was relatively small.
(iii) *Automation*
  The introduction of automated sample handling (Biomek i5 and i7 liquid handlers for transfer from collection tube to plate, lysis and heat inactivation and magnetic bead extraction of RNA, https://www.beckman.com/landing/ppc/liquid-handlers/biomek/i-series) reduced processing time by around 20% and reduced test failures 10-fold, from 5 to 0.5%, compared to manual testing.
(iv) *Addition of agents*
  One of the major challenges in the transition to automated testing of saliva samples was sample viscosity. The addition of 50 µL of 1M dithiothreitol (DTT, Sigma Aldrich) to 2 ml saliva plus stabiliser reduced sample viscosity and improved automated tube to plate transfer. Addition of DTT did not affect test sensitivity of either synthetic contrived or clinical SARS-CoV-2 samples.

### Study participation and methods

Informed consent was obtained from all participants within this internal SARS-CoV-2 testing program, consent was reviewed by the AstraZeneca Bioethics Advisory Board. This study was approved by the AstraZeneca internal review body with all methods performed in accordance with local relevant guidelines and standard operating procedures from AstraZeneca. Sourced samples for methods development were obtained according to AstraZeneca Human Biosamples policy, which includes review of informed consent and ethics approvals.
